# Development and properties of duplex MgF_2_/PCL coatings on biodegradable magnesium alloy for biomedical applications

**DOI:** 10.1371/journal.pone.0193927

**Published:** 2018-04-02

**Authors:** Preeti Makkar, Hoe Jin Kang, Andrew R. Padalhin, Ihho Park, Byoung-Gi Moon, Byong Taek Lee

**Affiliations:** 1 Institute of Tissue Regeneration, College of Medicine, Soonchunhyang University, Cheonan, South Korea; 2 Department of Regenerative Medicine, College of Medicine, Soonchunhyang University, Cheonan, South Korea; 3 Sk Innovation Global Technology, Daejeon, South Korea; 4 Advanced Metals Division, Korea Institute of Materials Science, Changwon, Gyeongnam, South Korea; Texas A&M University Baylor College of Dentistry, UNITED STATES

## Abstract

The present work addresses the performance of polycaprolactone (PCL) coating on fluoride treated (MgF_2_) biodegradable ZK60 magnesium alloy (Mg) for biomedical application. MgF_2_ conversion layer was first produced by immersing Mg alloy substrate in hydrofluoric acid solution. The outer PCL coating was then prepared using dip coating technique. Morphology, elements profile, phase structure, roughness, mechanical properties, *invitro* corrosion, and biocompatibility of duplex MgF_2_/PCL coating were then characterized and compared to those of fluoride coated and uncoated Mg samples. The *invivo* degradation behavior and biocompatibility of duplex MgF_2_/PCL coating with respect to ZK60 Mg alloy were also studied using rabbit model for 2 weeks. SEM and TEM analysis showed that the duplex coating was uniform and comprised of porous PCL film (~3.3 μm) as upper layer with compact MgF_2_ (~2.2 μm) as inner layer. No significant change in microhardness was found on duplex coating compared with uncoated ZK60 Mg alloy. The duplex coating showed improved *invitro* corrosion resistance than single layered MgF_2_ or uncoated alloy samples. The duplex coating also resulted in better cell viability, cell adhesion, and cell proliferation compared to fluoride coated or uncoated alloy. Preliminary *invivo* studies indicated that duplex MgF_2_/PCL coating reduced the degradation rate of ZK60 Mg alloy and exhibited good biocompatibility. These results suggested that duplex MgF_2_/PCL coating on magnesium alloy might have great potential for orthopedic applications.

## Introduction

Biodegradable metals have received huge attention in recent times due to their applications as orthopedic implants and cardiovascular interventional devices. They are attractive alternatives due to their higher load-bearing capacity and lower material cost compared to their polymeric counterparts [1–3]. Magnesium (Mg) and its alloys have unique ability of being degradable *in vivo* with density, modulus, and compressive yield strength close to human bone. Such closely matched mechanical properties can minimize stress shielding effects caused by difference in elastic modulus between foreign implant and host hard tissue [3–6]. However, non-homogenous and rapid corrosion with hydrogen gas release and increased alkaline pH in vicinity of implant have limited their clinical application [3–8].

Surface coating is a reliable and efficient method to improve corrosion resistance. A wide range of surface coatings such as conversion coatings, inorganic coatings, polymer coating, and combined coatings have been applied to increase corrosion resistance [7–9]. Among various anti-corrosion coating treatments, chemical conversion route is one of the simplest, effective, and economical processes [9]. Chemical conversion treatment method using hydrofluoric acid to form magnesium fluoride (MgF_2_) coating has been used to improve the corrosion resistance of potential degradable Mg based implants with good compactness, low water solubility, and high adhesive strength. In addition, it is easy to form coating uniformly on implants with complex shape and harmless release of fluorine ions into the organism [10–14]. For successful biomedical application of magnesium, an ideal coating should have improved corrosion resistance, good cell compatibility, and acceptable degradation rate of magnesium to meet healing requirement. In this regard, biodegradable polymer coating is a potential option [15–17]. Polycaprolactone (PCL) is a promising semi-crystalline aliphatic polymer widely used owing to its good biocompatibility, significant toughness and outstanding mechanical properties. PCL is a good candidate for bone tissue engineering because of its biocompatibility, bioresorbability, higher fracture energy and slow degradation rate [17–20]. Moreover, PCL is hydrophobic which can be desired for a coating on magnesium to control its high corrosion rate [7,18]. However, several disadvantages are associated with usage of PCL alone due to of lack of desired bioactivity and insufficient strength [21]. Thus, a combination of these two coatings on magnesium alloy surface might offer significant reduction for corrosion resistance with good biocompatibility.

A few studies have reported either focussing on MgF_2_ coating [10–14] or PCL coating [17–20] on magnesium alloy. However, the studies on the performance of PCL coatings with inner layer MgF_2_ on Mg substrate have not been reported yet. Thus, the objective of this study was to develop a novel duplex layered MgF_2_/PCL coating on magnesium alloy substrate by chemical conversion followed by dip coating method. The morphology, phase structure, chemical composition, roughness, wettability and performance in terms of mechanical properties, *invitro* degradation and biocompatibility of duplex MgF_2_/PCL coating onto magnesium alloy were evaluated and compared with single layered MgF_2_ coating and uncoated Mg alloy.

## Materials and methods

### Sample preparation

Commercial ZK60 magnesium alloy substrates with dimension of 10 mm x 10 mm x 2 mm were used in the present study. Its chemical composition (wt. %) is zinc (Zn) 5.5, zirconium (Zr) 0.49 and magnesium (Mg) balance. Prior to the coating deposition, substrates were first mechanically polished with silicon carbide papers upto 1200 grit to ensure the same surface roughness. Samples were then degreased with acetone for 10 min followed by rinsing with deionized water and dried in warm stream of air. For optical microstructure, ZK60 Mg alloy was polished further using 3 μm to 0.25 μm diamond paste. Etching solution was prepared by mixing 4.2 g of picric acid (98%, AppliChem GmbH, Korea) in a solution containing 10 ml of acetic acid (glacial, 99.5%, Samchun Pure Chemical Co., Ltd., Korea), 70 ml of ethanol (95%, Samchun Pure Chemical Co., Ltd., Korea) and 10 ml of deionized water. ZK60 Mg alloy was etched for 20–30 sec. The etched alloy was analyzed using optical microscopy (OM, BX51RF, Olympus, Tokyo, Japan) for their microstructural evaluation.

### Preparation of MgF_2_ and duplex MgF_2_/PCL coatings

For fluoride treatment, prepared samples were immersed vertically in a plastic bottle containing 48 wt. % hydrofluoric acid (HF, Sigma Aldrich, USA) at room temperature for 24 hr under constant stirring. Treated samples were then rinsed thoroughly with deionized water and air dried. Polycaprolactone (PCL, Mn-70000-90000, Sigma-Aldrich, USA) and dichloromethane (DCM, Sigma-Aldrich, USA) were used as starting reagents for deposition of polymer coating on the initial fluoride treated layer. A simple dip-coating technique was employed for PCL layer deposition. Briefly, PCL granules were dissolved in DCM solvent with magnetic stirring for 5 hr. HF treated substrates were then immersed in the prepared solution for 45 sec to allow wetting of the substrate. In order to obtain a stain-free surface, specimens were slowly and mechanically pulled out of the solution at a speed of 1mm/s. All coated samples were finally vacuum dried for 12 hr. All specimens were heated at 50°C for 10 min to remove moisture and entrapped air from substrate surfaces before immersing in the prepared PCL solution. The fluoride treated and duplex layered coatings were designated as MgF_2_ and MgF_2_/PCL coatings respectively in the following sections.

### Characterizations

#### Coating characterization

The surface morphologies of ‘as received’ ZK60 Mg alloy, MgF_2_, and duplex MgF_2_/PCL coatings were examined by scanning electron microscopy (SEM, JSM-635F, JEOL, Tokyo, Japan) equipped with an energy dispersive X-ray spectrometer (EDX, Oxford Instruments, UK). All samples were mounted onto aluminum stubs by using a double-sided carbon adhesive tape and sputter coated with carbon (Cressington 108 Auto, JEOL, Tokyo, Japan) prior to SEM imaging for conduction. The crosssection morphology of the duplex coatings was studied using transmission electron microscopy (TEM, 200 kV, F-20 (FEI)). TEM sample was prepared by focused ion beam (FIB) method. In order to protect PCL layer from gallium (Ga) ion beam, a layer of platinum (Pt ~1 μm) was deposited. Pt-deposited region was later trenched and lift out. To acquire the desired TEM slice, a current of 0.24 nA and 18 pA was employed for bulk milling and final thinning respectively.

Phases present in ZK60 Mg alloy, MgF_2_, and duplex MgF_2_/PCL coatings were determined using X-ray diffraction (XRD, D/MAX-250, Rigaku, Japan) with Cu Kα radiation. Diffraction patterns were recorded between 2θ values ranging from 20° to 80° with a scanning speed of 1°/min. Atomic chemical composition at samples surface was analyzed by X-ray photoelectron spectroscopy (XPS, Thermo Scientific MultiLab 2000, UK) using AlKα source with binding energies upto 1400 eV.

The surface roughness of uncoated and the coated alloy samples were examined by atomic force microscopy (AFM, XE-150, PSIA) in contact mode. The forces between a sharp probe (<10 nm) and surface area at distances (0–10 nm) were measured. The hydrophilicity of the samples was determined using contact angle measuring systems (DSA 100, KRUSS GmbH). The ultrapure water was dropped onto the samples surface with 10 μl drop volume and at dosing rate of 1 μl/sec. For each sample, an average of 7 readings was taken at different positions. Each sample set was carried out in triplicates.

Adhesion scratch test was carried out for single layered MgF_2_ and duplex MgF_2_/PCL coatings (three samples from each set) using a scratch tester (MST/NST, Anton Paar, US). A progressive load of 30–2000 mN was applied at a table speed of 60 mm/min with scanning length of 4 mm during the test. The hardness (HV) of ZK60 Mg alloy and duplex MgF_2_/PCL coatings was determined using Vickers microhardness tester (Mitutoyo HM-122, Germany) under a 10 gf load and dwelling time of 10 sec according to ASTM E384-17 standard. Each set of samples was repeated five times and their average value was calculated along with standard deviation.

#### *Invitro* degradation test

Immersion test was conducted to investigate the long-term stability of developed coatings on Mg alloy. The immersion tests of the uncoated, fluoride treated and duplex MgF_2_/PCL coatings were conducted according to the ASTM G31-72 standard following the protocol reported earlier [14,22]. Each set of ZK60 Mg alloy, MgF_2_, and duplex MgF_2_/PCL coated samples were immersed in 40 mL of phosphor buffer saline (PBS, Amresco, Korea, pH-7.4) solution in triplicates. The temperature was maintained at 37±0.5°C during the test. These immersed samples were extracted at different time intervals (1, 2, 3, 7, 10, and 14 d), gently rinsed with deionized water, and air dried. The weight of each sample set was measured prior to the test (W_0_). After the immersion test, the change in surface morphology and chemical composition of each sample was observed by SEM and EDX respectively. Before weight loss measurement, all samples were immersed in chromic acid solution (200 g/L Cr_2_O_3_ + 10 g/L AgNO_3_) for 5 min to remove the residual coatings and the corrosion products. Samples were then washed with deionized water, acetone, air dried and weighed (W_t_). The degradation rate of all samples was determined by measuring the relative %age weight loss of the samples according to the relation [22]:

$$W_{L}=[\frac{W_{t}-W_{0}}{W_{0}}]\times 100$$

where W_L_, W_0_, and W_t_ are the % age weight loss, and the weights before and after immersion, respectively. The variation of pH with time for each set of alloy samples was also studied. The pH value for each set of immersed samples was measured using a pH meter (Thermo Scientific, Korea) at a particular time interval for 14 days. PBS solution was replaced by fresh ones daily during the whole immersion test period. The amount of hydrogen release was tested by placing each set of substrates in PBS solution at 37°C under an inverted 15 ml tube. The solution level inside the 15 ml tube was measured intermittently for 14 days. Three samples were placed for each condition to measure hydrogen evolution for ZK60 Mg alloy, MgF_2_, and duplex MgF_2_/PCL coatings.

#### *Invitro* biocompatibility test

MC3T3-E1 preosteoblast cells (ATCC, CRL-2593, American cell bank, USA) were employed in the *invitro* cell culture experiment to ensure the biological compatibility of samples. They were cultured in a primary medium consisting of minimum essential medium (alpha-MEM, Gibco, USA), 10% fetal bovine serum (FBS, Aldrich, USA) and 1% penicillin-streptomycin antibiotic (PS, Bio-Whittaker, Arlington Heights, USA) in a humidified 5% CO_2_ atmosphere at 37°C. The cell viability of the samples was investigated by indirect cell assays (via extract) according to the protocols of ISO 10993–12. In indirect assay, MgF_2_ and duplex MgF_2_/PCL samples were initially placed in 12 well plates and sterilized with exposure of ultraviolent radiation on each side for 40 min. Samples was incubated with alpha-MEM (serum free) at 37°C for 24 hr. The supernatant solution from plates was then withdrawn, centrifuged for the 100% extract preparation which were diluted further into 12.5%, 25%, 50% concentrations with the primary medium for the cytotoxicity evaluation. MC3T3-E1 cells (2x10^5^) were preincubated in 96-well cell culture plates for 24 hr at 37°C to allow attachment. The culture medium was then replaced with different concentration of extracts and the medium was used as a blank control. The extracts along with cells were incubated for 1 day at 37°C with 5% CO_2_ atmosphere. 3-[4,5-dimethylthiazol-2-yl]-2,5-diphenyltetrazolium bromide (MTT, Gibco, CA) solution (100 μl/well) was then added and incubated at 37°C for 4 hr. At the end, 100 μl of dimethyl sulfoxide (DMSO, Samchun Pure Chemical, Korea) solution was added to each well. The sample plate was incubated for 1 hr and the absorbance value or the optical density (OD) of the resulting purple colored solution was measured by using an ELISA plate reader (EL 312, Biokinetics reader, Bio-Tek instrument) at wavelength 595 nm.

For cell proliferation studies, 2x10^5^ cells/well were seeded direct onto sterilized MgF_2_ and MgF_2_/PCL samples in 24 well plates. These samples were incubated at 37°C with 5% CO_2_ atmosphere for 1, 3, and 5 days. After a stipulated time, samples were washed thrice with PBS solution and proceed to fixing with paraformaldehyde (4%, Sigma Aldrich, USA) for 15 min at room temperature. The cells were later permeabilized with 0.5% Triton X-100 (Sigma Aldrich, USA) for 10 min. After subsequent blocking with 2.5% bovine serum albumin (BSA, Sigma-Aldrich, USA) solution for 60 min, cell membrane was immunostained with fluorescein isothiocyanate conjugated phalloidin (25 μl/ml FITC, Sigma Aldrich, USA) solution overnight at 4°C. Samples were later stained with 10 μg/ml Hoechst 33342 (Sigma Aldrich, USA) for 5 min for staining cell nuclei. The stained samples were finally washed with PBS solution thrice, mounted onto slides, and visualized under confocal fluorescent microscope (Olympus, FV10i-W, USA) along with the accompanied FV10i-ASW2.0 viewer software.

For cell adhesion studies, cell (2x10^5^) seeded samples were incubated for 4 hr in humidified atmosphere with 5% CO_2_ at 37°C. Following the same procedure steps until BSA blocking as mentioned above for proliferation, the cells were further immunostained using vinculin antibody (Millipore, US) and placed overnight at 4°C, followed by washing thrice with PBS. Afterwards, samples were incubated with Alexa Flour 594 (Invitrogen, US) conjugated secondary antibody for 1 hr at room temperature. At the end, cells were stained with FITC conjugated phalloidin and Hoechst 33342 at the specified interval, mounted and analyzed by confocal microscope.

#### *Invivo* test

To determine the effect of the duplex MgF_2_/PCL coating on the degradation behavior of the magnesium alloy, ZK60 Mg alloy and duplex MgF_2_/PCL coated samples were implanted into rabbits weighing approximately 3.0 kg. The care and use of laboratory animals were conducted according to the guidelines approved by the Animal Ethical Committee of Soonchunhyang University, South Korea. Animals were anesthetized with 3.0% isoflurane with oxygen gas pumped through a vaporizer (Harvard apparatus, USA). Fur on the right knee of the rabbit was shaved, the exposed skin was thoroughly cleaned with 70% ethanol and disinfected with povidone iodine solution. A full thickness incision measuring 3–4 cm in length was made along the lateral aspect of the knee to access the underlying joint. The distal femoral head was accessed by creating another incision on the overlaying soft tissues. The exposed distal femoral head was then drilled with a trephine drill with a 6 mm diameter at a height of 5 mm with constant irrigation with sterile saline solution. The implant (ZK60 Mg alloy or duplex MgF_2_/PCL) was then carefully placed within the bone defect. Control defect was left without any implant material. Three rabbits were used for each sample. All incisions were then closed with 0–5 Vicryl suture and disinfected with povidone iodine solution. Tramadol and Baytril were administered based on recommended doses to manage pain and as prophylaxis respectively. After 2 weeks, the animals were sacrificed via overdose of ethyl ether anesthetic. Tissue samples containing the implants were extracted and fixed using 10% formaldehyde solution. Extracted samples were then scanned with Skyscan 1076 microCT scanner (Bruker microCT, Kontich, Belgium). Data scans were reconstructed and analyzed to determine the progression of implant biodegradation and tissue development. Using CTAnalyzer software (Bruker microCT, Kontich, Belgium), the implanted sample was selected using a series of region of interest to create a volumetric selection. The data set was then segmented through two ranges of gray intensities to separate the implant and the surrounding bone tissue. Volume of the remaining sample was calculated using the same program. Three-dimensional reconstruction was created using the same corresponding data set using CTVox software (Bruker microCT, Kontich, Belgium).

The fixed tissues ZK60 Mg alloy, duplex MgF_2_/PCL were then dehydrated using subsequent series of ethanol then acetone immersion for 1 day each. After dehydration, the samples were infiltrated and embedded in methyl methacrylate (MMA) resin using benzoyl peroxide as a hardener. MMA embedded blocks were transversally cut using a diamond abrasive cutter (Topmet; R&B, Seoul, Korea) and were polished to 10–45 μm thickness. The slides were washed with distilled water and finally stained with Villanueva Osteochrome Bone stain. Micrographs of the stained tissue sections taken using an optical microscope (BX53 Olympus, USA) equipped with a DP72 digital camera.

## Results

### Surface characterization

**Fig 1:**
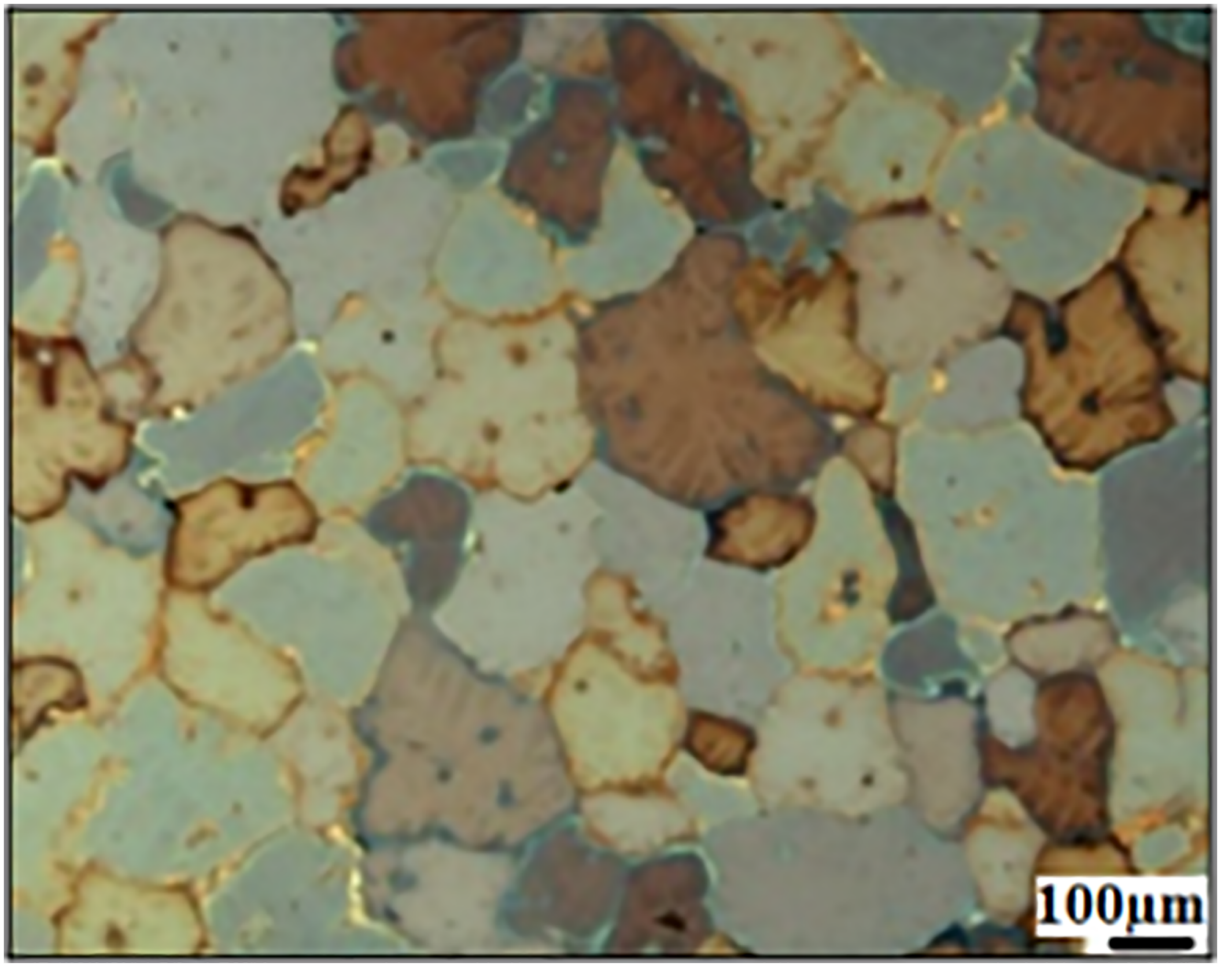
Optical micrograph of as-received ZK60 Mg alloy.

**Fig 2:**
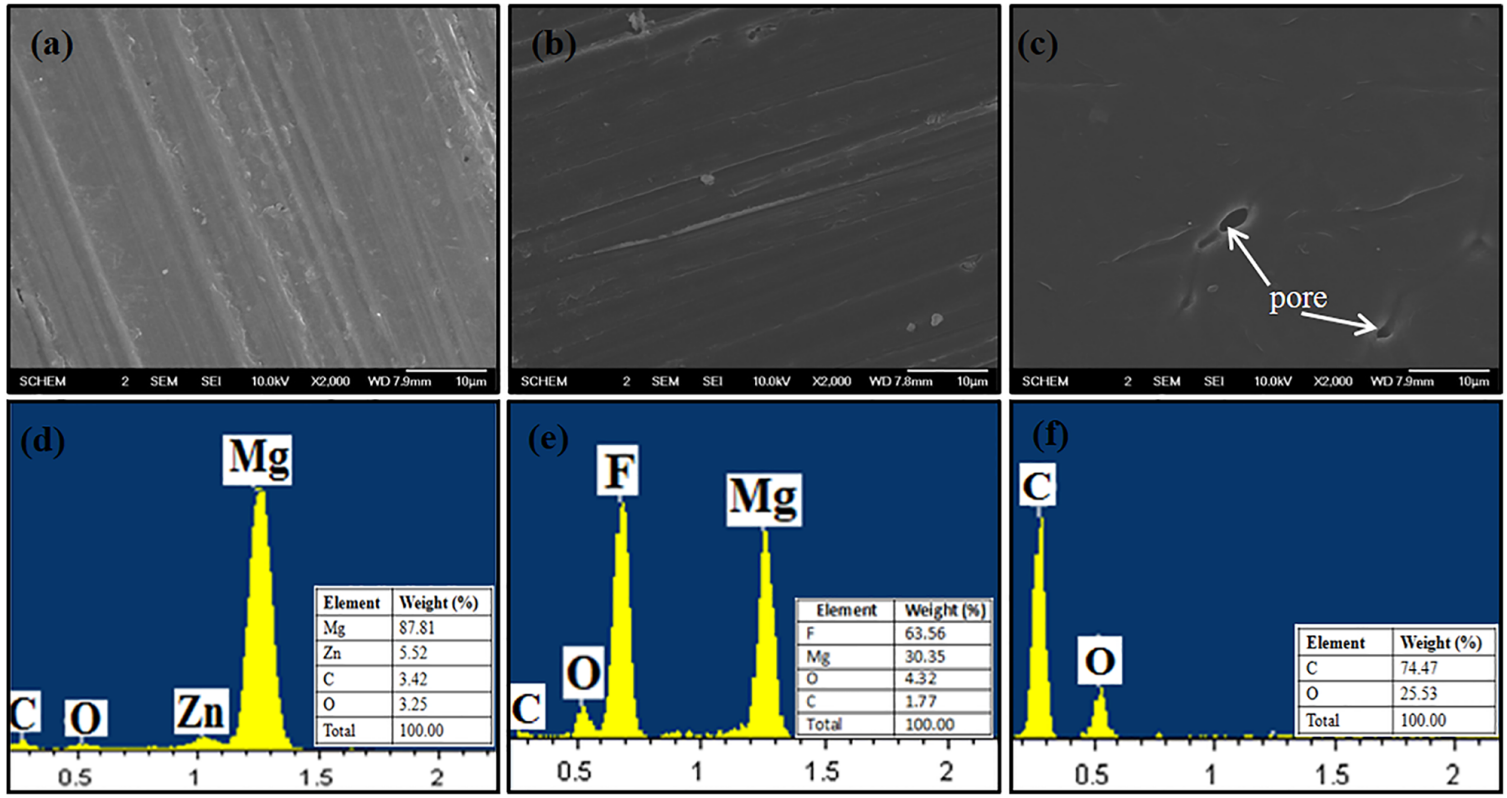
SEM images of (a) ZK60 Mg alloy, (b) MgF_2_ and (c) duplex MgF_2_/PCL coatings along with EDX (d-f).

Optical micrograph of as-received ZK60 Mg alloy is shown in Fig 1. The image revealed a courser and equiaxed grains of nearly 100–200 μm. It is also observed that some eutectics and precipitates of intermetallic MgZn phases were present within grains as well as along the grain boundaries (black spots). The eutectic constituted of a mixture of secondary Mg-Zn phase and supersaturated solid solution [23]. SEM micrographs and elemental compositions of ZK60 Mg alloy, surface modified MgF_2_, and duplex MgF_2_/PCL coatings are shown in Fig 2. The surface of as-polished ZK60 Mg alloy appeared to be slight course with abrasive vertical lines as shown in the SEM image (Fig 2a). After immersion in HF solution for 24 hr, the initial metallic grey color of ZK60 Mg alloy (Fig 2a) was uniformly changed to dark black (Fig 2b). Fluoride coated surface appeared to be completely covered by a compact and uniform film with few abrasive scratches occurred due to mechanical polishing. A transparent porous film was found on the fluoride treated surface throughout after PCL treatment (Fig 2c). The developed duplex MgF_2_/PCL coating was found to be uniform and crack-free (Fig 2c). Such morphological modification was supported by transformation of chemical composition for Mg, MgF_2_, and MgF_2_/PCL coatings. EDX analysis showed that magnesium (Mg) and zinc (Zn) elements were present in ZK60 Mg alloy with minute impurities of carbon (C) and oxygen (O) (Fig 2d). Quantitative analysis results were profoundly alternated after fluoride treatment and PCL coating. The presence of fluorine (F) apart from Mg and O confirmed the formation of MgF_2_ film on the substrate. The oxygen content might be attributed to the formation of Mg(OH)_2_. Minute traces of carbon are also seen in the film (Fig 2e). Small carbon content in both samples may arise due to the use of adhesive conductive carbon tape and carbon coating as required to avoid charging of sample’s surface during SEM imaging (Fig 2d and 2e). The presence of C and O elements in MgF_2_/PCL coatings indicated successful coating of PCL onto fluoride coated surface (Fig 2f).

**Fig 3:**
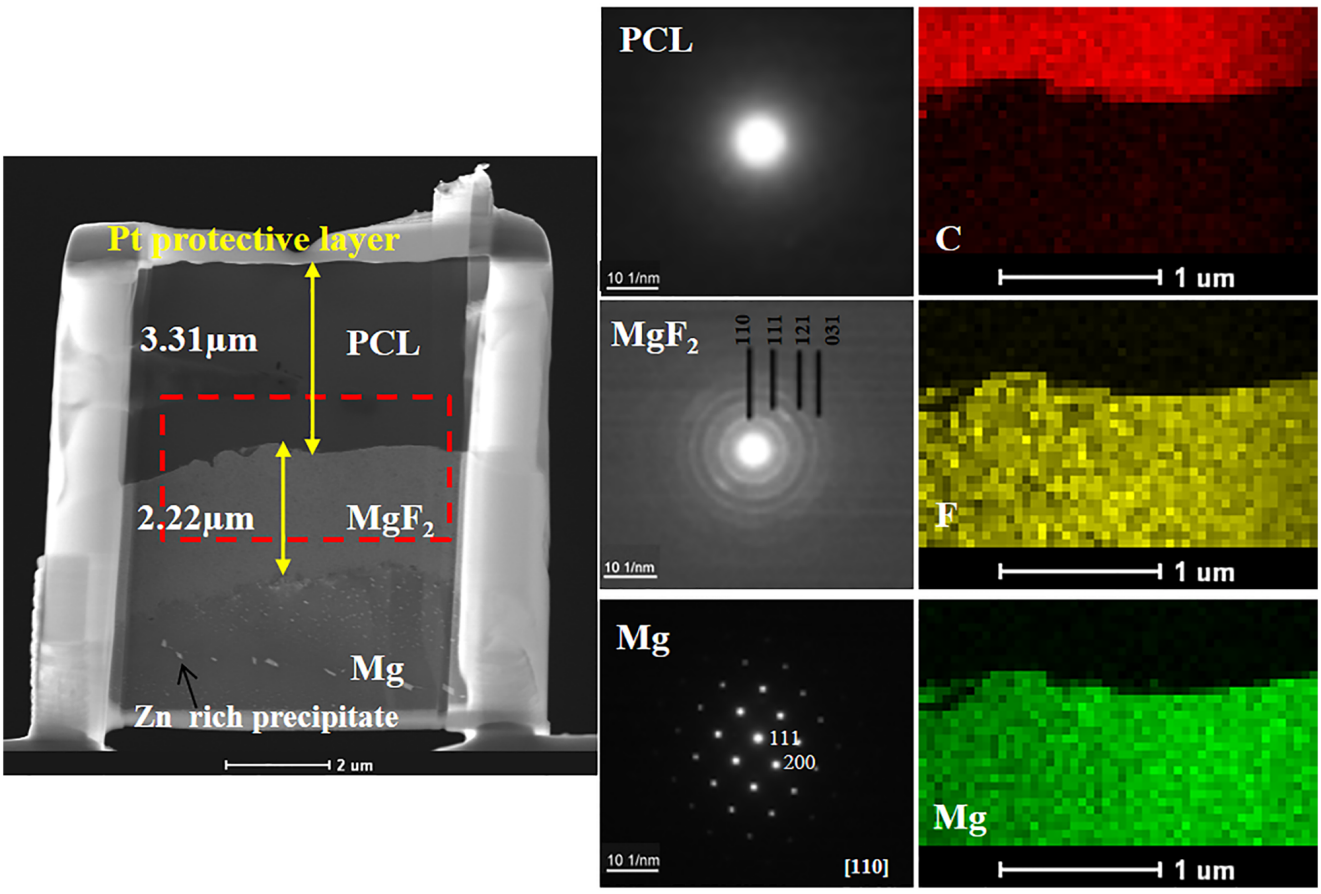
Dark field TEM image showing cross-section of duplex MgF_2_/PCL coatings along with corresponding SAD patterns and mapping profile.

**Fig 4:**
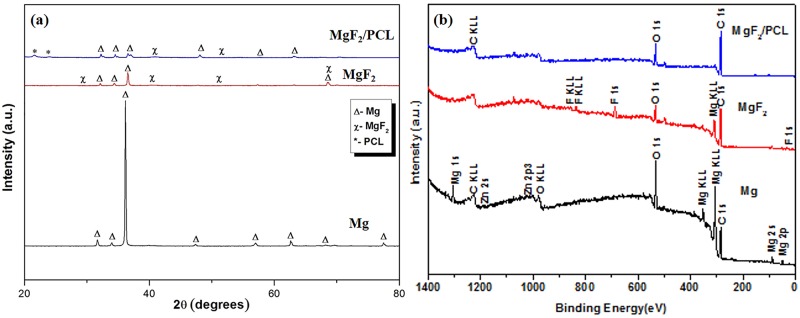
ZK60 Mg alloy, MgF_2_, and MgF_2_/PCL coatings (a) XRD, and (b) XPS analysis.

Crossectional morphology of duplex MgF_2_/PCL coating was investigated by TEM micrograph along with selected area electron diffraction (SAD) patterns and mapping profile. Results are shown in Fig 3. High angle annular dark field (HAADF) image showed clear interfaces of outer PCL, intermediate MgF_2_, and Mg substrate. The thickness of compact MgF_2_ film was approximately 2.2 μm and that of outer PCL coating was around 3.3 μm (Fig 3) regardless of surface irregularity. No defect or crack was observed at the interface of both coatings, showing evidence of good adhesion between the outer layer and the inner layer as well as between the inner layer and the Mg substrate. SAD patterns were obtained from each layer to confirm their crystallography. As expected, SAD pattern of ZK60 Mg alloy showed a hexagonal structure. However, in fluoride treated layer, MgF_2_ film had nanocrystalline nature with tetragonal structure (space group: P4_2_-mnm). The amorphous nature of PCL coating contributed to the diffused ring observed in SAD pattern (Fig 3). These results were in resonance with results of XRD studies (Fig 4a). X-ray mapping profile at the interface (red frame) displayed that C was mainly distributed at the outer layer whereas F became the main element in the inner layer besides Mg, confirming that the duplex coating was formed on the Mg substrate. Compositional analysis further confirmed homogenous distribution of both outer PCL and inner MgF_2_ coatings throughout the substrate.

XRD and XPS spectra of ZK60 Mg alloy, MgF_2_, and MgF_2_/PCL coatings are shown in Fig 3. X-ray diffraction pattern of as-received ZK60 Mg alloy confirmed Mg phase with hexagonal system (ICDD No: 01-071-6543) as shown in Fig 4a. After fluoride treatment for 24 hr, MgF_2_ (ICDD No: 01-070-8288) peak with tetragonal structure was primarily found along with Mg. However, the intensity of MgF_2_ peak was quite low compared to that of Mg, suggesting that the fluoride coating layer was thin as depth of CuKα line for Mg is around 0.8 μm at grazing angle of 1°C [12]. It is established that Mg can react with HF to yield MgF_2_ by displacement reaction and insoluble MgF_2_ can form a barrier coating on Mg [13,14]. Amorphous broad peaks of PCL were observed at 21°C and 24°C (ICDD No: 00-034-1796) apart from peaks of Mg and MgF_2_ in MgF_2_/PCL coatings (Fig 4a).

In order to fully understand the coating composition, chemical compositions of elements at the surface of Mg, MgF_2_, and MgF_2_/PCL coatings were investigated by XPS (Fig 4b). The chemical composition of ZK60 Mg alloy consisted of Mg and Zn with high concentration of C and O. Carbon in XPS surface scan is commonly attributed to environmental impurities due to extraneous hydrocarbons. Oxygen rich layer might have originated from hydrocarbons or due to formation of MgO and Mg(OH)_2_ on exposure to air [10]. After pretreatment with HF, an additional peak of fluorine in XPS spectra was observed, signifying the deposition of MgF_2_ film on the substrate. Moreover, binding energy of F1s at fluoride coated surface was around 685 eV, which was correctly matched to MgF_2_ value i.e. 685 eV with reference to standard database [24]. Also, the binding energy of O1s at around 533 eV could be ascribed to existence of small amount of Mg(OH)_2_ which is also evident in EDX (Fig 2e) [25]. After PCL modification, an increase in C1s and O1s without characteristic peaks associated with Mg and F confirmed complete coverage of PCL coating on MgF_2_ film [21]. XPS results were in consistent with the conclusion obtained from EDX data (Fig 2d–2f). Both XRD and XPS studies verified that PCL was successful anchored on HF treated surface.

**Fig 5:**
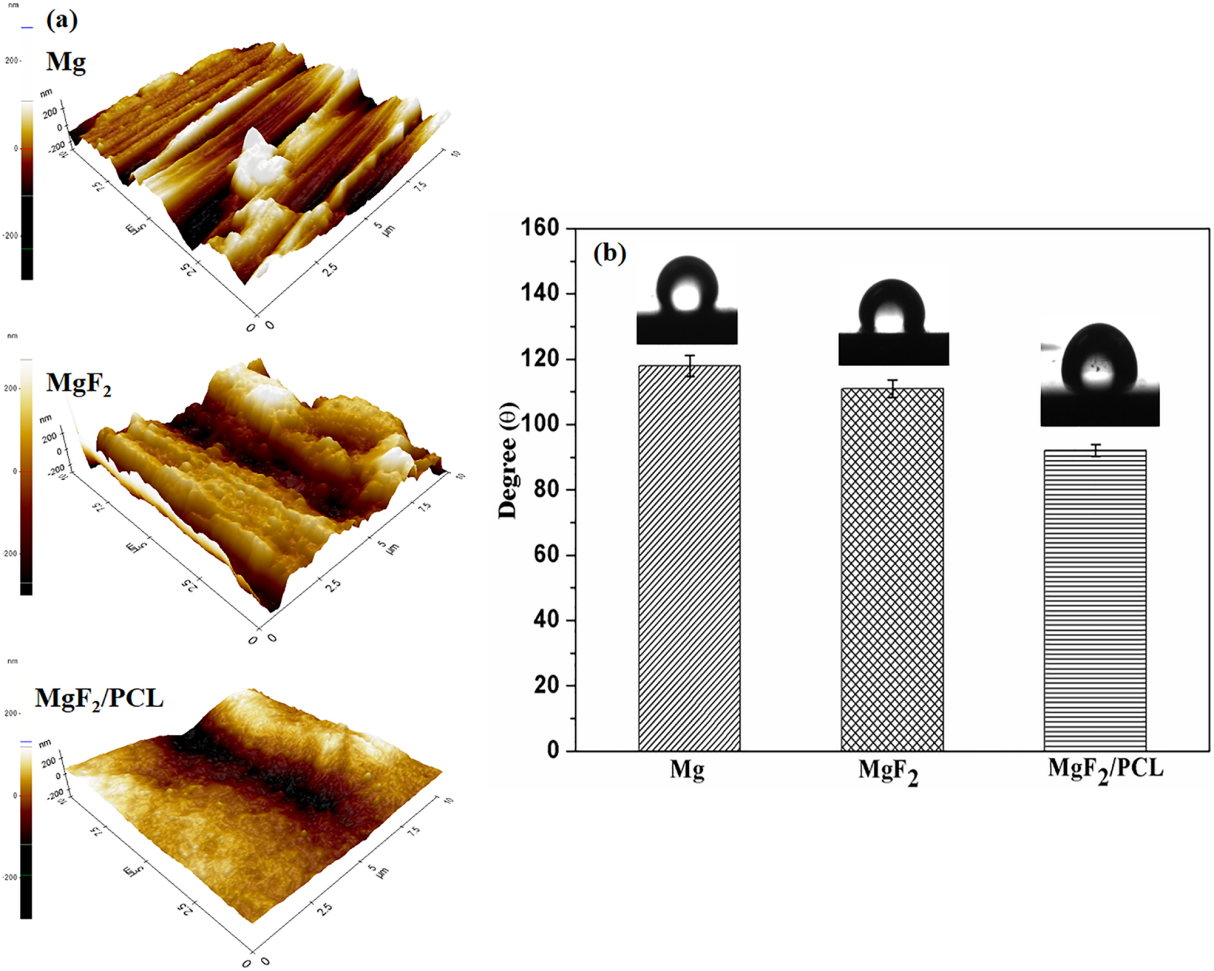
ZK60 Mg alloy, MgF_2_ and MgF_2_/PCL coatings (a) AFM, and (b) Wettability studies.

Three-dimensional AFM images of ZK60 Mg alloy, MgF_2_, and MgF_2_/PCL coatings are shown in Fig 5a. The average roughness value (Ra) of ZK60 Mg alloy was found to be 40.7 nm. All of the coated surfaces were found to be relatively rougher than uncoated ZK60 Mg alloy. The roughness value after fluoride (MgF_2_) coating was significantly increased to 66.3 nm. Minute pores generated on the surface due to acid immersion might be ascribed to the above increment. The surface morphology was described by the precipitation of intermetallic particles (MgZn) on the surface and at grain boundaries (Fig 1). Hydrogen released at cathodic sites could facilitate the emergence of pores in the resulting film. Similar results with coarser conversion coating were reported in the literature [14]. The duplex PCL coated surface was relatively smooth than MgF_2_ film and the average roughness value was found to be 49.8 nm. Surface roughness has also huge impact on corrosion resistance of samples. Reduction in surface roughness can lead to decreased surface area for corrosive attack [26]. Also, surface roughness is the pivotal factor that influencing biocompatibility. It has been reported that coatings with surface roughness in an optimum range of 20–100 nm can promote cell adhesion and longevity [27].

Photographs of water droplets on the surface of uncoated Mg and coated MgF_2_ and MgF_2_/PCL samples and variation in their wettability are shown in Fig 5b. Contact angles of ZK60 Mg alloy, MgF_2_, and MgF_2_/PCL coatings were 118 ± 3.2°, 111 ± 2.7°, and 92 ± 1.8°, respectively. These results indicated that both coatings increased the surface hydrophilicity compared to uncoated substrate. An increase in hydrophilicity could enhance adhesive interaction between water drop and the surface, thus decreasing contact angle. The lower contact angle can strongly support cell adhesion, spreading and proliferation [28].

### Mechanical characterization

**Fig 6:**
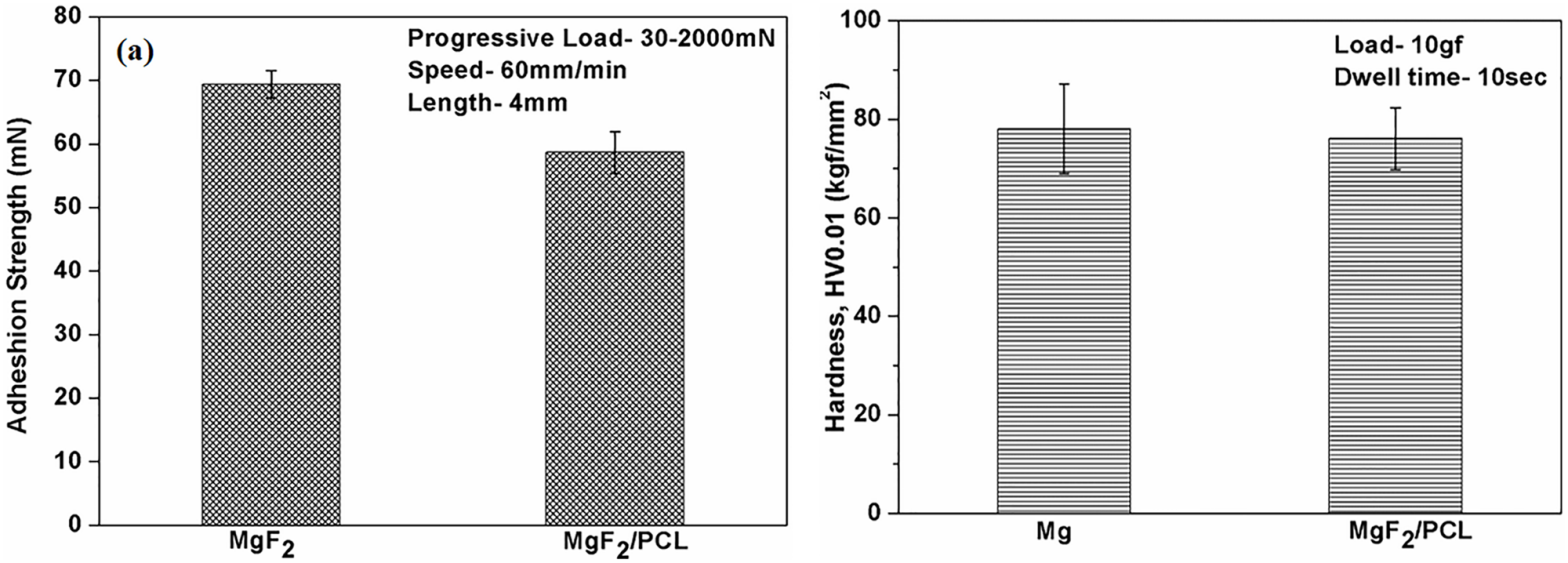
(a) Adhesion strength of MgF_2_, PCL and MgF_2_/PCL coatings, and (b) Hardness studies of ZK60 Mg alloy and duplex MgF_2_/PCL coatings.

The bonding strength between the coating and substrate plays a crucial role in determining the reliability as well as performance of the coated substrate as an effective implant material [29]. Scratch adhesion test was performed to evaluate the adhesion strength of MgF_2_ and MgF_2_/PCL coatings on ZK60 Mg alloy (Fig 6a). For comparison, adhesion strength of plane PCL coating on ZK60 Mg alloy was also investigated (Fig 6a). Adhesion strength values of single layered MgF_2_, PCL and duplex MgF_2_/PCL coatings were found to be 65.39± 2.2 mN, 42.5 ± 1.2 mN and 58.68 ± 3.27 mN respectively. These results indicated that both MgF_2_ film and MgF_2_/PCL coatings was strongly adhered to the substrate compared to PCL coated substrate. Xu et al. (2012) studied that PCL film exhibited poor bonding strength with substrate which might be due to its molecular structure. PCL possess lower ratio of oxygen by its weight, which leads to weak electrostatic interaction with Mg substrate [20]. Also, the bulging of PCL coatings was reported in literature after 7 days of exposure to corrosive environment during an immersion test [30]. It was clearly seen that the adhesion strength between PCL and substrate could be improved through inner layer MgF_2_ coating. The relatively increased adhesion of MgF_2_/PCL coatings over PCL coatings was more likely due to the mechanical interlocking as PCL solution was infused into MgF_2_ film sealing the micro pores and thus forms a stronger bonding layer with MgF_2_ film. In addition, the higher interfacial adhesion strength of MgF_2_ film with the PCL might be due to the formation of hydrogen bonding between -OH group present in fluoride treated surface and = O group of PCL [31]. The chemical composition of MgF_2_ film showed the existence of O along with F elements as evident from EDX (Fig 2e) and XPS (Fig 4b) analysis which might contribute OH^-^ ions on its surface.

Vickers microhardness values of uncoated and duplex MgF_2_/PCL coating are shown in Fig 6b. The average microhardness of ZK60 Mg alloy and the duplex MgF_2_/PCL coatings was found to be 78 ± 9 kgf/mm^2^ and 76 ± 6 kgf/mm^2^ respectively. The results revealed that hardness was not significantly changed between uncoated and duplex coated samples. It is reported that the PCL coating is not efficient for enhancing the strength of the substrate [32,33]. It is also established that PCL coatings on Mg alloy decreased the degradation rate while sustaining the bulk mechanical properties on degradation [17,34]. This indicates that duplex MgF_2_/PCL coating might be able to withstand higher loads, thus offering better corrosion resistance to Mg substrate [35].

### *Invitro* degradation studies

**Fig 7:**
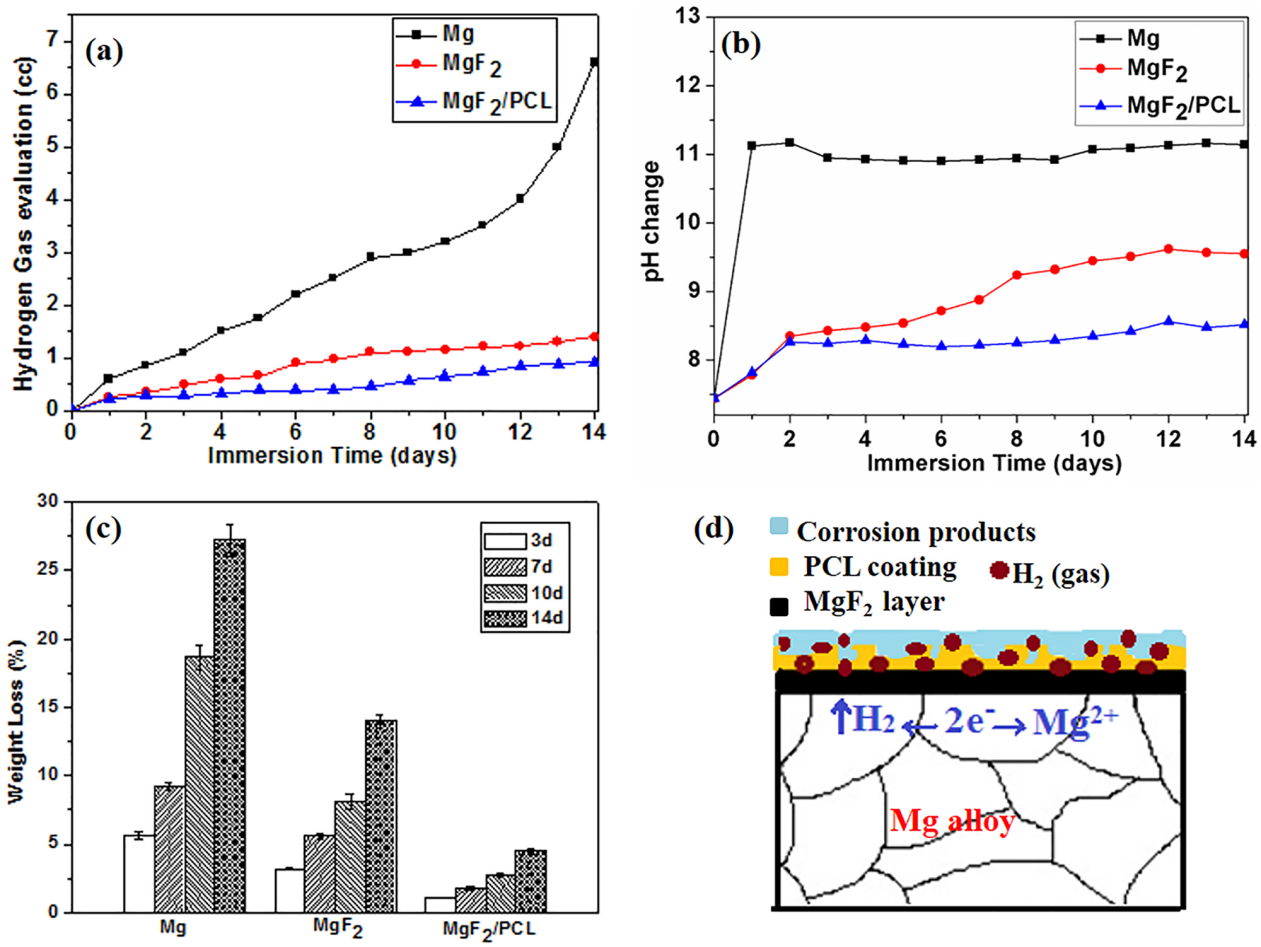
(a) pH change, (b) Volume of hydrogen released, (c) Weight loss as a function of immersion time in PBS solution for 14 days for ZK60 Mg alloy, MgF_2_, and MgF_2_/PCL coating samples. (d) Schematic illustration of the degradation mechanism of MgF_2_/PCL coated alloy after immersion in PBS solution.

**Fig 8:**
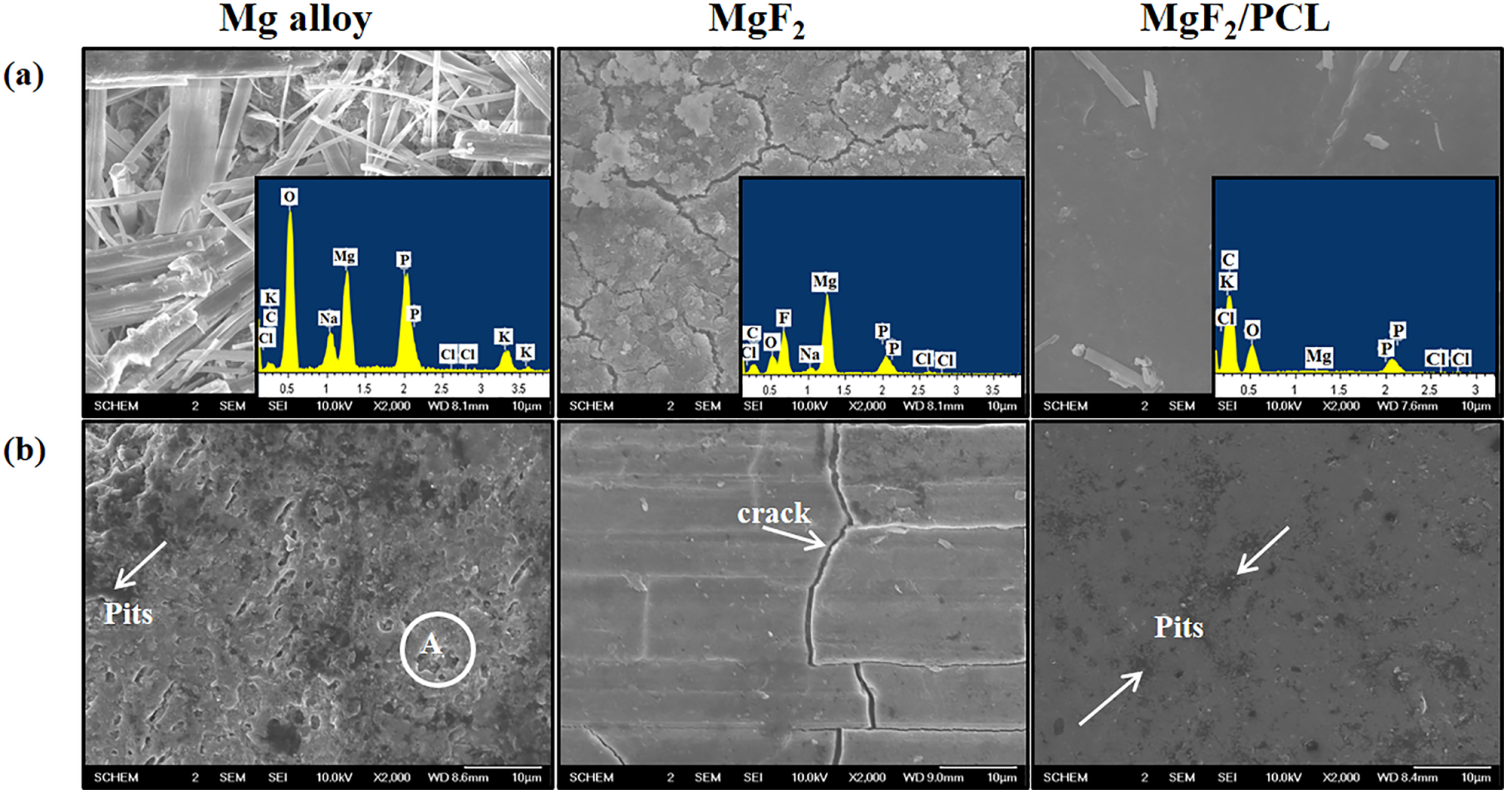
SEM images of ZK60 Mg alloy, MgF_2_, and MgF_2_/PCL coatings. (a) After immersion in PBS solution for 14 days along with EDS; (b) After washing of corroded products.

The primary goal of surface modification is to delay initial degradation rate or enhance corrosion resistance of biodegradable magnesium [36]. It was well-known that the corrosion resistance of the coated substrates was influenced by various factors like thickness, microstructure and composition of the surface coating [37]. Fig 7 shows the variations in pH change, hydrogen evolution and weight loss for ZK60 Mg alloy, MgF_2_, and MgF_2_/PCL coatings after immersion in PBS solution at 37°C for 14 days. Variation in pH as a function of time for different samples immersed in PBS solution is shown in Fig 7a. The pH value of duplex MgF_2_/PCL coatings was initially increased for 3 days followed by relatively stable behavior afterwards. In contrast, the pH value of ZK60 Mg alloy was first increased intensely within 1 day. It then showed an almost constant behavior (Fig 7a). After 14 days of incubation, the pH values of Mg, MgF_2_, and MgF_2_/PCL were 11.07, 9.45, and 8.35, respectively. The pH values for both MgF_2_ and MgF_2_/PCL were found to be lower than that of ZK60 Mg alloy. This suggested that the protective outer layer of PCL and the relatively compact inner layer of MgF_2_ effectively controlled the degradation of magnesium substrate. The less significant increase of pH values of MgF_2_/PCL coated substrates could be attributed to the release of alkaline ions. It is apparent that the presence of an inner layer of MgF_2_ eases the local alkalization and lessens the acidification of the medium as polymer (PCL) degrades to acids [38]. However, the pH values of the samples after a long incubation time (after 2 days) were mainly dependent on degradation of Mg substrate. The increase in pH of MgF_2_ could be possibly due to more rigorous destruction of MgF_2_ coating than that of duplex MgF_2_/PCL coating. Hence, Mg substrate with MgF_2_ coating was more degraded compared to that with MgF_2_/PCL coating at later stage. The reduction in long-term protective ability of MgF_2_ coating might be due to the existence of micro-cracks in the coating evident in SEM micrographs shown in Fig 8b. These results indicated that the prominent alkalization effect on ZK60 Mg alloy was effectively reduced by surface coatings. In other words, duplex MgF_2_/PCL coating could effectively prevent the penetration of immersion fluids (Fig 7a).

Hydrogen gas evolution plots for the Mg, MgF_2_ and MgF_2_/PCL coated samples in the PBS solution for 14 days are shown in Fig 7b. Results showed that the volume of released hydrogen was significantly reduced in MgF_2_ and MgF_2_/PCL coated samples compared to that in ZK60 Mg alloy during the immersion period. The result was resonant with the pH change trend (Fig 7a). Duplex MgF_2_/PCL coatings exhibited the lowest amount of hydrogen evolved (Fig 7b). It was revealed that the ZK60 Mg alloy had experience a severe corrosion whereas coated MgF_2_ and MgF_2_/PCL samples could efficiently inhibit the degradation in the PBS solution. These results suggest that duplex coatings can offer higher corrosion resistance compared to ZK60 Mg alloy or fluoride treated samples.

Fig 7c shows the average weight loss of ZK60 Mg alloy, coated MgF_2_ and MgF_2_/PCL coating in PBS solution for time periods of 3, 7, 10, and 14 days incubated at 37°C. It was observed that MgF_2_ coated samples displayed effectively lower weight loss rate with respect to Mg substrate. The rate of weight loss, as expected, was further declined due to the duplex layer (MgF_2_/PCL) protection on the ZK60 Mg alloy which retards the corrosion rate. ZK60 Mg alloy exhibited the highest weight loss rate among other samples. An increasing trend in rate of weight loss was noticed for all the samples with increase in exposure time. The % weight loss of coated MgF_2_ and MgF_2_/PCL samples were about 13.6% and 4.5%, respectively compared to ZK60 Mg alloy (21.2%) after 14 days of immersion. The continuous increment in weight loss of coated samples with prolonged exposure time indicated the delamination of the coating layers on Mg substrate. It signified that both MgF_2_ and MgF_2_/PCL coatings were not providing adequate protection for longer immersion time. In addition, it was found that the corrosion rate was more accelerated in the initial 3 days of exposure compared to prolonged immersion time (14 days) for ZK60 Mg alloy (Fig 7c). This could be explained by the fact that initially larger surface area was exposed to solution which in turn promote the exothermic reactions and thus enhanced the corrosion rate. The higher concentration of chlorine and other salts might also contribute to the increased corrosion rate in early few days. On the other hand, the corrosion rate of both MgF_2_ and MgF_2_/PCL coated samples notably increased with exposure time due to the detachment or weak adhesion of the protective layers after longer immersion in the PBS solution. Immersion tests revealed that duplex MgF_2_/PCL coated alloy experienced lesser corrosion attack compared with single layered MgF_2_ coating and uncoated alloy samples.

Fig 7d displays the schematic diagram showing degradation mechanism of duplex MgF_2_/PCL coated alloy after immersion in PBS solution. When duplex coated samples were immersed in PBS solution, it is likely that PBS penetrated from the porous structure of the PCL coatings to the ZK60 Mg alloy substrate via MgF_2_ layer. As early the corrosive PBS medium reached the Mg substrate, the immediate galvanic reactions between primary Mg and the secondary phases took place. As a result, the following anodic and cathodic reactions occurred [6,7]:

$$Mg\to Mg+2e^{-}\;\left(Anodic\;reaction\right)$$ (1)

$$2H_{2}O+2e^{-}\to H_{2}+2OH^{-}\;\left(Cathodic\;reaction\right)$$ (2)

$$Mg+2H_{2}O\to Mg{\left(OH\right)}_{2}↓+H_{2}\left(g\right)↑$$ (3)

$$Mg{\left(OH\right)}_{2}↓+2Cl^{-}\to MgCl_{2}+2OH^{-}$$ (4)

As per the reactions, Mg metal from substrate was converted into insoluble magnesium hydroxide Mg(OH)_2_ film with evolution of hydrogen gas (H_2_) (Eq (3)). Hence, Mg(OH)_2_ starts assembling at the alloy-coating interface and resulted in pH increase of the solution. Also, the evolved H_2_ gas pushed away the coated PCL film which in turn weakened the adherence between the PCL coating and ZK60 Mg alloy. Apart from loosening of film, the gas accumulation below the coating leads to crack formation and other failures in the coating. At the same time, due to the presence of aggressive salts such as chloride ions in solution could damage the protective PCL layer by converting Mg(OH)_2_ into soluble MgCl_2_ (Eq (4)). Hence, the PCL film on the surface would be readily dissolved leading to the generation of pits. The nucleation of pits on the surface of MgF_2_/PCL coatings after immersion in PBS for 14 days is evident in SEM image (Fig 7b). Also, the depletion of Mg(OH)_2_ layer and utilization of OH^-^ ions by the corrosion products released in PBS solution further speed up the solution penetration into the ZK60 Mg alloy resulting in increase in the corrosion rate.

SEM images of surfaces of ZK60 Mg alloy, MgF_2_, and MgF_2_/PCL coating after immersion in PBS solution for 14 days along with EDX are shown in Fig 8a. It can be clearly seen that ZK60 Mg alloy was fully covered with corrosion products. On the other hand, fluoride treated sample, MgF_2_, showed network-like cracks with small precipitates evenly distributed over the entire surface of the alloy. The thicker corroded layer might be ascribed to the presence of micro-cracks in the coating caused by the shrinking of surface corrosion product during drying. However, a few white precipitates were observed on duplex MgF_2_/PCL coated surface. EDX analysis of corroded surface compositions for different samples showed the presence of O, C, Na, K, Cl, and P elements. The existence of elements suggested the formation of MgCl_2_, Mg(OH)_2_ and other Na and K rich compounds in the corrosion product. Therefore, contents of these elements on the surface after immersion can be used to estimate the degree of corrosion occurred in each sample. It was observed that ZK60 Mg alloy had much more corrosion products on its surface than MgF_2_ or MgF_2_/PCL coated sample. Duplex coated surface showed significantly lower concentrations of P and O with the absence of K or Na compared to ZK60 Mg alloy. The lower concentration of Mg and higher concentration of C in MgF_2_/PCL coated sample indicated that PCL coating was still present after 14 days of immersion. However, in case of MgF_2_, traces of Na and P with relatively higher concentration of Mg depicted the destruction of fluoride coating on Mg substrate (Fig 8a). SEM images of washed corroded surface are shown in Fig 8b. ZK60 Mg alloy surface showed a large number of cracks, small as well as deep corrosion pits (region A) with severe degradation on the surface. The crack formation may account to the surface shrinkage and dehydration of the corrosion products [39]. Fluoride treated surface showed slightly severer corrosion by the presence of micro-cracks compared to MgF_2_/PCL coated sample after 14 days of immersion. The cracks in MgF_2_ coating can provide a suitable path for penetration of PBS solution into the coating (Fig 8b). Hence, corrosion expands under the coating along with the corrosion products that might result in weak adherence of the coating and eventually lead to coating failure [40]. Duplex coatings showed small pits on its PCL surface (Fig 8b). All corrosion surface images were consistent with degradation results during the whole immersion period of 14 days. It is well known that MgF_2_ is more stable than MgO and Mg(OH)_2_ and efficient in resisting corrosion attack [10]. Hence, in duplex MgF_2_/PCL coating systems, outer PCL layer retard the corrosion attack while the inner MgF_2_ layer prevent the ZK60 Mg alloy substrate from aggressive medium when it crosses from the outer PCL layer. These results suggested that duplex MgF_2_/PCL coated sample exhibited higher corrosion resistance compared to MgF_2_ coating or ZK60 Mg alloy.

### *Invitro* biological studies

**Fig 9:**
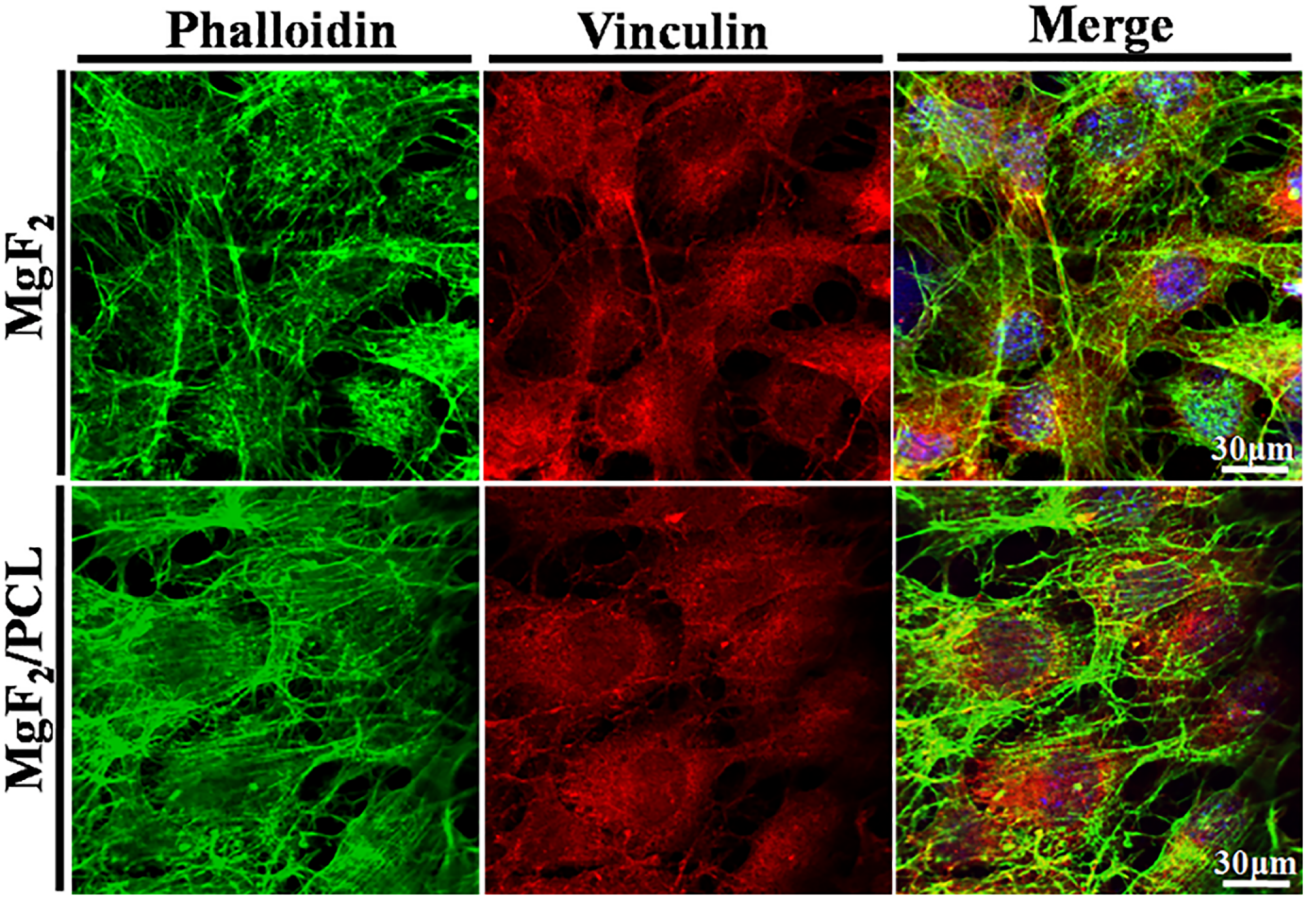
Confocal images of adhesion and attachment of MC3T3-E1 cells after 4 hr seeding on MgF_2_ and MgF_2_/PCL coatings. Phalloidin, Vinculin and nucleus were labelled with green, red and blue respectively.

The degree of proliferation and adhesion of host cells on the substrate specifies its biocompatibility [41]. Cell adhesion behavior of MC3T3-E1 cells on MgF_2_ and MgF_2_/PCL coatings after 4 hr of incubation is shown in Fig 9. It was observed that cells were well attached onto the surfaces of both coatings. Furthermore, staining for focal adhesion and the actin cytoskeleton was carried out where Phalloidin, Vinculin and nuclei were detected as green, red and blue respectively. The strongly developed cytoskeletal structure and interconnected cellular network was noticed for both the coatings. It seemed that MgF_2_/PCL coatings exhibited higher cell attachment and more spreading than MgF_2_ coating.

**Fig 10:**
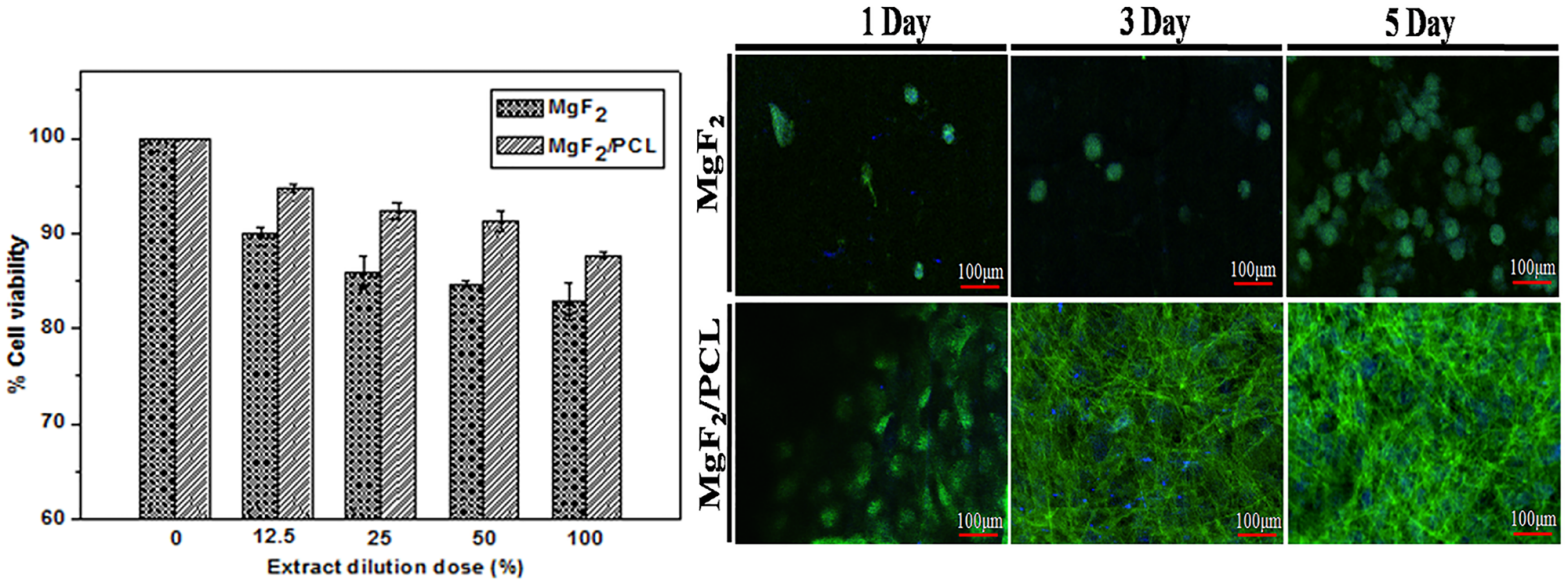
(a) Cytotoxicity of MgF_2_ and duplex MgF_2_/PCL against MC3T3-E1 cells. MTT assay was performed using indirect assay after 1day incubation with increasing extract concentrations. (b) Cell proliferation behaviour of MgF_2_ and MgF_2_/PCL coating after incubation of 1, 3, and 5 days.

Results of cell viability studies of MgF_2_ and MgF_2_/PCL coatings using indirect cell assay with increasing extract concentrations after 1 day of incubation are shown in Fig 10a. When extract concentration was increased from 12.5% to 100%, the % of cell viability was decreased from 90% to 82% for MgF_2_ and 95% to 87% for MgF_2_/PCL coatings. MgF_2_/PCL coatings showed higher cell viability than MgF_2_ coating at all extract concentrations.

Quality proliferation of the MC3T3-E1 cells on the MgF_2_ and MgF_2_/PCL coating were examined by confocal imaging (Fig 10b). The images demonstrate that cells attached and continuously proliferated on both the coatings. Both coatings surface supports the cells growth and its metabolic activity. It was revealed that the cell density was the highest for the MgF_2_/PCL coating after 5 days in culture. The proliferation results showed that more cells seemed to be attached onto MgF_2_/PCL than MgF_2_ coating after 1, 3 and 5 days of incubation. It is also indicated that the MgF_2_/PCL coating had higher cell density and more cell spreading compared to MgF_2_ coating after incubation for 1, 3, and 5 days. The proliferation data correlated with the pH results showing that MgF_2_ coating might influence the significant cell growth and morphology as pH increase continuously with time (Fig 7b). PCL coating on fluoride treated Mg substrate might cause topographical simulation and boost focal cell adhesion on virtue of its higher hydrophilicity compared to MgF_2_ and ZK60 Mg alloy (Fig 5b). The cell proliferation and cell adhesion results were in good agreement with cell viability showing that MgF_2_/PCL coatings support cell growth and proliferation and exhibited better biocompatibility.

### *Invivo* studies

**Fig 11:**
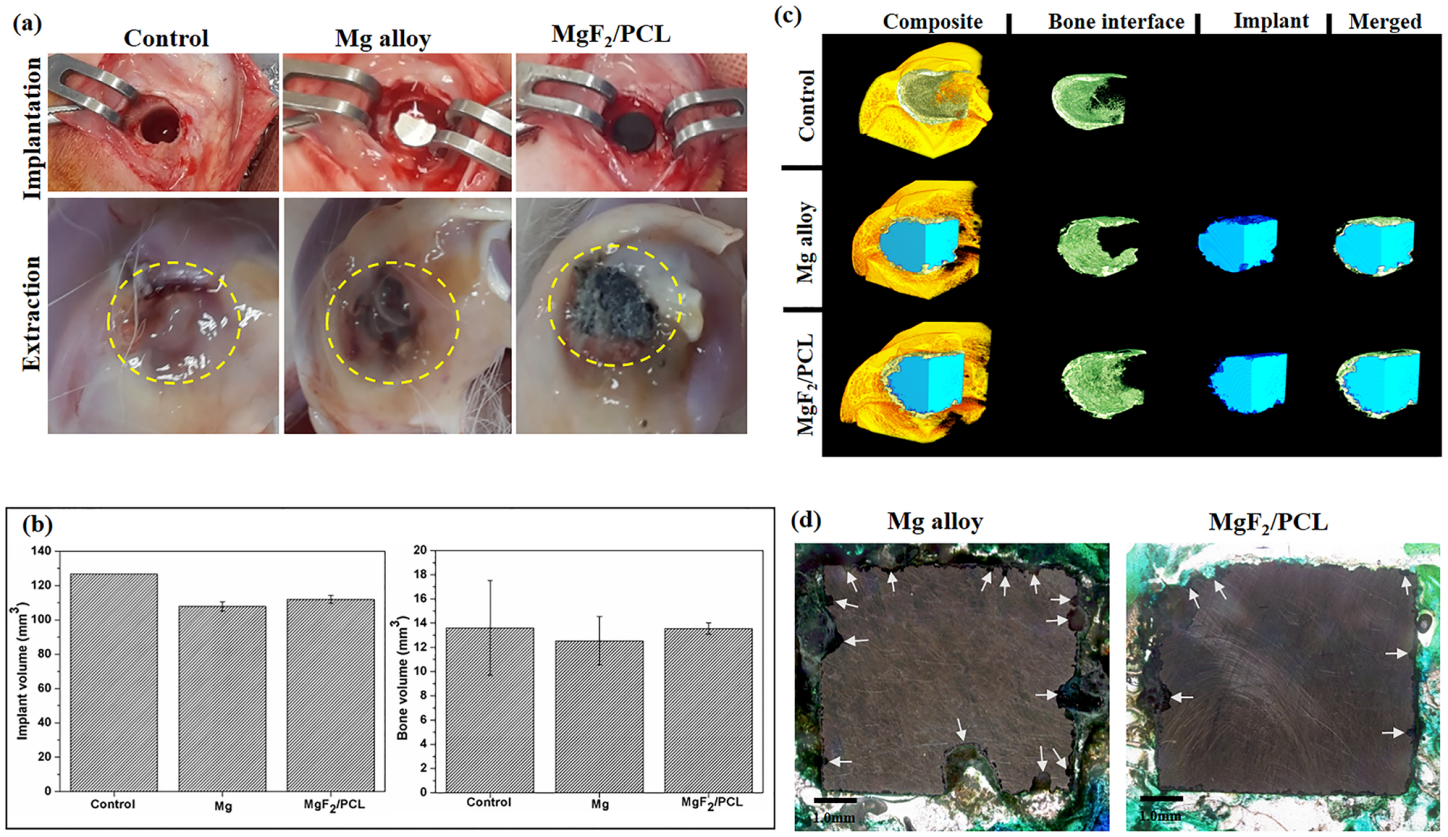
Control (defect), ZK60 Mg alloy and duplex MgF_2_/PCL coating (a) Photographs at the time of implantation and extraction, (b) Degradation rate and Bone volume at the interface, (c) 3-D MicroCT scans of the implanted zone and (d) Histological analysis after 2 weeks implantation.

Results of *invivo* studies conducted on ZK60 Mg alloy and duplex MgF_2_/PCL coating using rabbit model are shown in Fig 11. During implantation, both ZK60 Mg alloy and duplex MgF_2_/PCL coatings did not show significant reaction upon contact with blood or bone tissue (Fig 11a). Photographs of the surgical sites prior to extraction of bone tissue containing the implants were also taken (Fig 11a). A subcutaneous gas accumulation was noticed around ZK60 Mg alloy whereas no subcutaneous bulging or gas cavity was observed around duplex MgF_2_/PCL coatings after 2 weeks of implantation (Fig 11a). It was probably due to massive hydrogen gas generation and magnesium ion release in the vicinity of ZK60 Mg implant. Apart from that, a moderate amount of accumulated interstitial fluid was seen on the Mg alloy implant site upon extraction. It might be related to the buildup of degradation products which lead to the inflammatory response of the surrounding tissue. No signs of swelling or inflammation was found on duplex MgF_2_/PCL coating upon extraction. Control (defect) shows gradual reduction of the inflammatory phase after 2 weeks (Fig 11a).

Micro-computed tomography was generally used for proper visualization of the corrosion morphology and quantification of *invivo* corrosion rate [34]. Fig 11b represents the degradation rate and the interfacial bone formation of ZK60 Mg alloy and duplex MgF_2_/PCL alloys after 2 weeks. Analysis of the microCT data showed approximately 15.94±2.14% volume reduction for ZK60 Mg alloy while duplex MgF_2_/PCL coating revealed about 11±1.76% volume reduction (Fig 11b). The results indicated that the duplex coating was capable of reducing the magnesium ions release in *invivo* environment. The lower degradation of duplex MgF_2_/PCL coating was attributed to the protective effect of the duplex coating. However, it seemed that slight corrosion was initiated in duplex coating under *invivo* conditions. Higher pores in PCL provide a suitable path for penetration of body fluid and make contact with the inner MgF_2_ layer which might get destructed during 2 weeks. It was reported that high molecular weight PCL coating exhibited *invivo* higher corrosion rate compared to low molecular weight PCL on account of increased pores and larger pore size [17]. The inner destructed MgF_2_ layer leads to the direct exposure of the ZK60 Mg alloy to the body fluid. The corrosion rate of the Mg alloy would be dramatically increased at these sites because of the galvanic effect between coated Mg alloy and the exposed Mg alloy substrate [42]. As a result, the severe local corrosion would have occurred at these sites. The under layer corrosion products formed would exert a pressure that might lifts the outer PCL coating and eventually some part of PCL coating was detached from the Mg alloy substrate before being completely degraded [34]. Even though corrosion occurred on ZK60 Mg alloy and MgF_2_/PCL samples, the interfacial bone formation was still found around the implants [34,43]. Three dimensional (3-D) analysis of bone formation along the implant interface indicated no significant difference between control (13.61±4.91 mm^3^), ZK60 Mg alloy (12.34±1.99 mm^3^) and duplex MgF_2_/PCL coating (13.56±0.47 mm^3^) within 2 weeks (Fig 11b). However, the bone formation appeared to be more consistent in duplex MgF_2_/PCL coating compared to ZK60 Mg alloy implant. It may be ascribed to the mild hydrogen gas generation and lower magnesium ions release as duplex coating reduced the direct contact of substrate with the body (Fig 11b and 11c). According to literature, the release of low magnesium ions enhances the osteoblastic activity and hence induce a stimulatory effect on the new bone tissue growth [17,43]. The results of the volumetric analysis were further exemplified upon viewing the 3-D reconstructions of the scanned tissue samples (Fig 11c).

Closer examination of the tissue sections revealed a difference in progression of implant degradation between ZK60 Mg alloy and duplex MgF_2_/PCL coating implants. Histological analysis of ZK60 Mg alloy and duplex MgF_2_/PCL coating after Villanueva osteochrome bone staining for 2 weeks are shown in Fig 11d. ZK60 Mg alloy showed larger amount of deep pitting corrosion sites (arrow) along the edges in contact with the surrounding tissue signifying the higher dissolution rate of the alloy. On the other hand, duplex MgF_2_/PCL coating showed a relatively lesser pitting corrosion depicting slower degradation at 2 weeks. Formation of the gas cavities in the subcutaneous tissue around the ZK60 Mg alloy implant caused by hydrogen generation is a direct evidence of the rapid corrosion (Fig 11a). The result was in agreement with the volumetric analysis conducted from the microCT scans suggesting that duplex MgF_2_/PCL coating showed lower degradation rate than that of ZK60 Mg alloy. Taken together, the results of *invitro* and *invivo* studies revealed that the duplex MgF_2_/PCL coating exhibited lower degradation and good biocompatibility compared to ZK60 Mg alloy for 2 weeks.

## Conclusions

Duplex MgF_2_/PCL coating was successfully prepared on biodegradable magnesium alloy in this study. It comprised of an inner layer of MgF_2_ film and an outer layer of PCL film deposited by chemical conversion and dip coating methods respectively. Morphology, phase and chemical composition studies revealed the presence of PCL coating (~3.3 μm) and MgF_2_ film (~2.2 μm) onto ZK60 Mg alloy. Duplex MgF_2_/PCL coatings showed better adhesion strength than PCL coatings. No significant change was found in the microhardness of duplex MgF_2_/PCL coating when compared to ZK60 Mg alloy. Hydrogen evaluation, pH change, and degradation tests showed that the duplex protective layer of MgF_2_/PCL exhibited an enhancement in corrosion resistance compared to single layered MgF_2_ or ZK60 Mg alloy. The barrier properties of uniform PCL film along with compact MgF_2_ film contributed to lowest corrosion rate. Such duplex MgF_2_/PCL coating also supported cell viability, cell adhesion and proliferation better than MgF_2_ coated and uncoated alloy sample. *Invivo* studies indicated that duplex MgF_2_/PCL coating reduced the degradation rate of ZK60 Mg alloy and exhibited good biocompatibility for 2 weeks of implantation. These results indicated that outer PCL coating might be a promising approach after fluoride treatment. However, long-term *invivo* studies are required to further validate the application of duplex coated implant for orthopaedic applications.
